# A Novel Multi-Ingredient Supplement Activates a Browning Program in White Adipose Tissue and Mitigates Weight Gain in High-Fat Diet-Fed Mice

**DOI:** 10.3390/nu13113726

**Published:** 2021-10-22

**Authors:** Joshua P. Nederveen, Katherine Manta, Adam L. Bujak, Alexander C. Simone, Matthew R. Fuda, Mats I. Nilsson, Bart P. Hettinga, Meghan C. Hughes, Christopher G. R. Perry, Mark A. Tarnopolsky

**Affiliations:** 1 Department of Pediatrics, Faculty of Health Sciences, McMaster University Medical Centre (MUMC), 1200 Main St. W, Hamilton, ON L8N 3Z5, Canada; nedervj@mcmaster.ca (J.P.N.); mantak@mcmaster.ca (K.M.); simona6@mcmaster.ca (A.C.S.); fudam@mcmaster.ca (M.R.F.); 2 Exerkine Corporation, McMaster University Medical Centre (MUMC), 1200 Main St. W, Hamilton, ON L8N 3Z5, Canada; adam.bujak@exerkine.com (A.L.B.); mats.nilsson@exerkine.com (M.I.N.); bart.hettinga@exerkine.com (B.P.H.); 3 Muscle Health Research Centre (MHRC), School of Kinesiology & Health Science, York University, 4700 Keele Street, Toronto, ON M3J 1P3, Canada; meghanh@yorku.ca (M.C.H.); cperry@yorku.ca (C.G.R.P.)

**Keywords:** mitochondria, obesity, multi-ingredient supplement, browning, high-fat diet, antioxidant

## Abstract

We investigated the effects of a novel multi-ingredient supplement comprised of polyphenol antioxidants and compounds known to facilitate mitochondrial function and metabolic enhancement (ME) in a mouse model of obesity. In this study, 6-week-old male C57/BL6J mice were placed on a high-fat diet (HFD; ~60% fat) for 6 weeks, with subsequent allocation into experimentalgroups for 4 weeks: HFD control, HFD + ME10 (10 components), HFD + ME7 (7 components), HFD + ME10 + EX, HFD + EX (where ‘+EX’ animals exercised 3 days/week), and chow-fed control. After the intervention, HFD control animals had significantly greater body weight and fat mass. Despite the continuation of HFD, animals supplemented with multi-ingredient ME or who performed exercise training showed an attenuation of fat mass and preservation of lean body mass, which was further enhanced when combined (ME+EX). ME supplementation stimulated the upregulation of white and brown adipose tissue mRNA transcripts associated with mitochondrial biogenesis, browning, fatty acid transport, and fat metabolism. In WAT depots, this was mirrored by mitochodrial oxidative phosphorylation (OXPHOS) protein expression, and increased in vivo fat oxidation measured via CLAMS. ME supplementation also decreased systemic and local inflammation markers. Herein, we demonstrated that novel multi-ingredient nutritional supplements induced significant fat loss independent of physical activity while preserving muscle mass in obese mice. Mechanistically, these MEs appear to act by inducing a browning program in white adipose tissue and decreasing other pathophysiological impairments associated with obesity, including mitochondrial respiration alterations induced by HFD.

## 1. Introduction

Over the last three decades, obesity prevalence has nearly tripled and continues to rise, with nearly one third of the global population classified as overweight and ~10% being clinically obese [1]. In the United States alone, ~70% adults aged 20 and older are overweight, with ~40% being clinically obese [2]. This growing epidemic in both developed and emerging countries [3] is particularly worrisome considering that obesity is related to many pathological conditions including fatty liver disease, type 2 diabetes, musculoskeletal disorders, several cancers, cardiovascular diseases, osteoarthritis, decreased mobility, and poor mental health [4,5].

Excessive weight gain is multifactorial in nature, with many hormones, cytokines, and neuropeptides being involved in the regulation of energy intake and expenditure [6,7]. During weight gain, chronic energy surplus results in the expansion of the white adipose tissue (WAT) through the storage of triglycerides in adipocytes, which causes an acute hypoxic environment in WAT, leading to the infiltration of macrophages and the subsequent release of pro-inflammatory cytokines from WAT [8]. Similarly, excessive energy intake causes substrate overload and oxidative stress in mitochondria, leading to mitochondrial dysfunction [9]. Obesity is associated with reduced mitochondrial biogenesis, oxidative respiration, and mitochondrial gene expression [10,11]. Concurrent with mitochondrial dysregulation is an increase in mitochondrial ROS production [12] and a subsequent decrease in intracellular antioxidants, leading to oxidative stress and abnormal cytokine production [13]. In this way, obesity and mitochondrial dysfunction dysregulate the production of WAT-derived circulating adipokines such as interleukins (IL)-6, IL-1β, and tumor necrosis factor (TNF)-α, which may exacerbate the state of low-grade, chronic inflammation observed in obesity [14]. Taken together, obesity is a multifactorial disease with roots in mitochondrial dysfunction, upregulated ROS production, and dysregulation of WAT-derived secretory factors. 

A key physiological process that has become a critical target for the enhancement of metabolism is the ‘browning’ or ‘beiging’ of white adipose tissue [15]. Beige white adipose tissue refers to the appearance of the tissue as it takes on more mitochondria, leading to greater lipolysis, lower inflammation, and lower oxidative stress [16]. 

Fundamentally, obesity is caused by an imbalance in energy intake versus expenditure [17]. The current first-line treatment for weight loss can include medications acting centrally on the brain to promote feelings of satiety [18], increasing energy expenditure through physical activity, and lowering dietary energy intake. However, these medications can come with side effects [19], and lifestyle recommendations are often met with poor adherence [20]. Collectively, these issues combined with the high prevalence of obesity have led to renewed consideration of pharmaceutical or nutraceutical approaches targeting energy intake, nutrient absorption, or energy expenditure [11]. A number of nutraceutical compounds have been shown to mitigate several of the aforementioned obesity-associated pathologies, including mitochondrial dysfunction, oxidative stress, and impaired lipolysis [21,22,23,24,25,26,27,28,29,30].

Therefore, the purpose of this study was to investigate whether supplementation of multiple targeted nutraceuticals could increase energy expenditure, enhance mitochondrial biogenesis and/or function, ameliorate ROS production, lower systemic inflammation, and ultimately increase WAT browning/fat oxidation in high-fat diet-fed animals, mitigating weight gain. Given that exercise prescription is part of both first-line obesity therapy and general weight maintenance, there are theoretical concerns that any nutraceutical approach containing antioxidants could attenuate the metabolic benefits of exercise [31,32]. We thus also examined whether supplementation would attenuate the benefits of endurance exercise.

## 2. Materials and Methods

### 2.1. Ethical Approval and Dietary Interventions

Ethics approval was granted by the McMaster University Animal Research Ethics Board and conformed to the standards of the Canadian Council on Animal Care (AUP#16-05-15). Male C57/BL6J diet-induced obesity mice were ordered from Jackson Laboratories (Bar Harbor, ME, USA) and placed on a high-fat diet (HFD) containing 60% energy from fat at 6 weeks of age, with the exception of chow (CHOW) control animals. All animals (total *n* = 178) were provided with food and water ad libitum and were housed in separate standard microisolator cages with enrichment (12 h light/dark cycle at 22 °C) to accurately record food consumption.

At ~12 weeks of age, mice were allocated into experimental groups which were standardized by body weight (BW). For ~1 month, the experimental groups were fed 1 of the following HFDs containing ~60% energy from fat: HFD control (HFD), HFD containing mitochondria-enhancing agents, HFD containing weight loss agents, or HFD containing a combination of both mitochondria and weight loss-enhancing agents or a standard chow diet. All diets are listed in Supplementary Table S1. In brief, mitochondria-enhancing agents included beetroot extract, coenzyme Q10 (CoQ10), alpha lipoic acid (α-LA), vitamin E, and creatine [21,22,23,24,25,28,33]. Weight loss agents included green tea extract (GTE), black tea extract (BTE), green coffee bean extract (GCBE), conjugated linoleic acid (CLA), and forskolin [27,29,30,34,35]. To demonstrate the effects of a healthy and/or standard diet on body composition in sedentary conditions, a group of mice were fed chow diet throughout (CHOW). To evaluate the effects of exercise (EX) and combination therapy (HFD with nutritional supplementation and exercise), animals were housed in cages containing an exercise wheel and underwent structured exercise (3 days/week).

### 2.2. Preliminary Study to Examine Effect of Supplementation (Experimental Approach 1)

To determine which dietary composition and/or intervention was able to reduce BW and fat mass (FM) most effectively, we examined animals (*n* = 10/group, standardized pre-intervention by BW) provided with either the control or experimental diet, with or without exercise. Prior to the introduction of the experimental diets, baseline testing was conducted on all animals. Baseline testing included the following measurements: BW, relative FM and lean mass (LM), muscle strength, and maximal running capacity. During the intervention, food consumption and BW were measured. All anthropometric, organ weight, food consumption, and exercise performance data for this preliminary experiment are listed in the appended supplemental data (Figure S1A–H). We determined that the composition comprised of both mitochondria-enhancing agents and weight loss agents (ME10) provided the most robust changes in body composition and weight, the effect of which was enhanced with exercise (ME10 + EX). These changes were more robust than when a composition of mitochondrial-enhancing agents or, separately, a composition of weight loss agents were provided (Figure S1A) to the animals. These results led to the next experimental approach (see below). 

### 2.3. Study to Determine the Effect of Combinatory Supplementation (Experimental Approach 2) 

To verify that the dietary composition was able to: (i) effectively reduce BW and FM, (ii) enhance exercise-mediated improvements, (iii) evaluate an ME with fewer ingredients (ME7), and (iv) evaluate potential biological mechanism(s) for the observed weight loss, we repeated the experiment. We examined an independent cohort of animals (*n* = 12/group, standardized by BW). The control HFD, CHOW, and ME10 diets were identical to those of Study 1, as were the exercise groups (EX, ME10 + EX). To define the minimal, essential components of the ME10 supplement driving the observed weight loss in the preliminary study, we eliminated BTE, CLA, and creatine, and termed this 7-component supplement ME7. The ME7 group received HFD containing only 3 of the weight loss agents (forskolin, GTE, GCBE) and only 4 of the mitochondria-enhancing agents (BE, CoQ10, Vit E, α-LA), removing creatine, BTE, and CLA (Supplementary Table S1). 

### 2.4. Metabolic Measurements In Vivo Following Short-Term Feeding (Experimental Approach 3)

To evaluate the effects of ME7 and ME10 supplementation on fat oxidation and basal energy expenditure compared to HFD control following a short-term period of supplementation, we performed metabolic monitoring using the Comprehensive Lab Animal Monitoring System (CLAMS; Columbus Instruments), with lipid oxidation rates collected via indirect calorimetry and activity levels measured in beam-breaks across the *x*-axis of the metabolic cages similar as previously described [36]. C57/BL6 (*n* = 24) diet-induced obesity mice were ordered from Jackson Laboratories and provided the same HFD (HFD) at 6 weeks of age. At ~12 weeks of age, mice were allocated into groups (*n* = 8/group) standardized by BW, placed in the metabolic cages, and allowed to acclimate to the new environment for ~12 h. Following the acclimation period, mice remained on the HFD control diet for a period of 1 day (baseline; BSL) to collect metabolic data for each group (prior to any change in diet). After 1 day of measurements (BSL) on the HFD control, mice were administered HFD, HFD + ME7, or HFD + ME10 diets for a 3-day period. Diet was removed from the animals prior (as they slept) to sacrifice on the third day of experimental diet provision. 

### 2.5. Food Consumption Tracking

At the onset of the study and at an age of 6 weeks, 65 g of the designated diet was provided to each cage. During the experimental period the feed weight was measured, ensuring that feed was removed from both the hamper and cage bedding. Food consumption was calculated as the difference in total feed weight over 24 h. When the feed reached 25 g, an additional 40 g of feed was added to ensure there was always sufficient food. 

### 2.6. In Vivo Measures of Adiposity and Relative Lean Mass

Relative FM and LM were quantified using a time-domain nuclear magnetic resonance (NMR) whole-body composition analyzer (Minispec LF90II, Bruker; Billerica, MA, USA) and normalized to body weight. 

### 2.7. Maximal Running Capacity Exercise Test

Animal exercise capacity was measured at baseline and endpoint via endurance stress test performed on a treadmill. All animals were placed in individual treadmill lanes and allowed to acclimate for 5 min prior to the initiation of the endurance stress test. The exercise session was preceded by a 10-min warm-up at 10 m/min. Following this period, the speed was increased by 1 m/min until animals had reached exhaustion. Exhaustion was determined at the point when the animals remained unresponsive to hind limb stimulation via paintbrush for >5 s. The speed and time of exhaustion of each individual animal was recorded.

### 2.8. Structured Endurance Exercise Intervention

To address the mitigation of obesity through exercise [37], endurance exercise training was performed by select groups (EX, ME10 + EX). These groups performed structured endurance exercise on an Exer 6 M treadmill (Columbus Instruments, Columbus, OH, USA) for 45 min/session, 3 days/week (alternate days) for the duration of the study to complement nightly cage wheel running activity. Exercise sessions were preceded by the same 5-min acclimation and 10-min warm-up at 10 m/min as the endurance stress test. After initial familiarization, animals were run at a speed of 15 m/min. Animals were encouraged to run on the treadmill with gentle hind-limb stimulation via paintbrush when they stopped running. All animals completed all exercise bouts. The animals in these groups were independently housed in voluntary running wheel cages and utilized the running wheels freely.

### 2.9. Animal Euthanasia

Mice were briefly anesthetized with isoflurane (Abraxis Bioscience, Los Angeles, CA, USA). Animals were euthanized via exsanguination, with blood being collected for downstream analysis. The tibialis anterior (TA) muscles, the quadriceps (left quadricep; SkM), liver, inter-abdominal epididymal white adipose tissue (eWAT), and brown adipose tissue (BAT) were all weighed, harvested, and snap-frozen using liquid nitrogen. The right quadricep was prepared for high-resolution respirometry. Snap-frozen samples were then placed in −80 °C freezer for long-term storage. For Study 3, tissues were harvested under isoflurane sedation to maximize the detection of the phosphorylation status of AMP-activated protein kinase (AMPK) [38].

### 2.10. Mitochondrial Respiration in Permeabilized Muscle Fiber Bundles

Muscle fibers were permeabilized similarly to previously described [39]. High-resolution O_2_ consumption measurements were conducted in MiR05 buffer using the Oroboros Oxygraph-2k (Oroboros Instruments, Corp., Innsbruck, Austria). In brief, we examined ADP-stimulated respiration, with pyruvate and malate added as complex I substrates (via generation of NADH to saturate electron entry into complex I), followed by a titration of submaximal ADP and maximal ADP. To examine complex I + II state 3 respiration, this was followed by addition of succinate (PMDG + S). Complex II kinetics were separately determined through the stepwise addition of succinate in the presence of ADP and rotenone (to inhibit and prevent superoxide generation at complex I). The detailed methods can be found in the Supplementary Experimental Procedures.

### 2.11. Immunoblotting

Tissues were prepared and homogenized in Lysing Maxtrix D tubes (MP Biomedicals, Solon, OH, USA) via the FastPrep-24 Tissue and Cell Homogenizer (MP Biomedicals) in ice-cold RIPA lysis and extraction buffer (ThermoFisher Scientific, Waltham, MA, USA, 89901) supplemented with protease and phosphatase inhibitor cocktail (Halt^TM^, ThermoFisher Scientific, 78440). Protein concentrations were then determined using a BCA Protein Assay Kit (Pierce, Thermo Scientific, 23225). Samples were subsequently prepared in Laemmli Buffer (Fisher Scientific, AAJ61337AC). Immediately prior to electrophoresis, samples were heated at 95 °C for 5 min and centrifuged, unless they were used for mitochondrial complex expression analyses. These samples were heated to 37 °C, followed by centrifugation. Samples were then loaded on a 4–20% Criterion TGX Precast Midi Protein Gel (Bio-Rad Laboratories, Hercules, CA, USA 5671094) with a PageRuler Plus Prestained Protein Ladder Protein Ladder at 10 to 250 kDa (Thermo Fisher, 26619), and run in 1X Tris/Glycine/SDS buffer (Bio-Rad Laboratories, 1610732). Following electrophoresis, samples were transferred using Trans-Blot Turbo Midi Nitrocellulose Transfer Packs (Bio-Rad Laboratories, 5671094) and stained with Ponceau S solution (Sigma, P7170-1L) for 5 min. The membranes were imaged and placed in blocking solution (5% BSA in 1X TBS) for 1 h, prior to overnight primary antibody incubation at 4 °C. All primary antibodies were prepared in 5% BSA in TBS. The antibodies were the OXPHOS (oxidative phosphorylation) cocktail, SOD1, SOD2, 4-HNE, UCP1, AMPK total protein, and phosphorylated AMPK (Thr172), and were used at a concentration of 1:1000 (information in Supplementary Table S2). The membranes were washed in 1X TBST and incubated with the appropriate secondary antibody (Jackson Immunoresearch 715-035-151, 715-035-152) at 1:10,000 (5% BSA in 1X TBS) for 1 h. The membranes were washed again in 1X TBST, then incubated with Clarity Western ECL substrate (Bio-Rad Laboratories, 170-5061) containing 50% luminol/enhancer and 50% peroxide solution for 5 min. Membranes were developed using ChemiDoc MP Imaging System (Bio-Rad Laboratories). Band intensities were quantified using Image Lab 6.1 software (Bio-Rad Laboratories) and normalized to Ponceau S staining. 

### 2.12. RNA Isolation and cDNA Synthesis

RNA was extracted from tissue, following lysis in Trizol Reagent (Life Technologies, Burlington, ON, Canada), into Lysing Maxtrix D tubes (MP Biomedicals, Solon, OH, USA) using the FastPrep-24 Tissue and Cell Homogenizer (MP Biomedicals). The RNA phase was subsequently purified using the PureLink™ RNA Mini Kit (Thermo Scientific, cat. no. 12183025) as per the manufacturer’s instructions. RNA concentrations (ng⋅mL^−1^) and purity (260/280) were determined with the Nano-Drop 1000 Spectrophotometer (Thermo Fisher Scientific, Waltham, MA, USA). Samples were then reverse-transcribed using a high-capacity cDNA reverse transcription kit (SuperScript^®^ VILO™ Master Mix; Invitrogen, Waltham, MA, USA, cat. no. 11755050).

### 2.13. Real-Time Quantitative PCR (RT-qPCR)

The absolute abundance for specific mRNA transcripts was assayed using TaqMan^®^ Fast Advanced Master Mix (cat. no. 4444963) and TaqMan Gene Expression Assays purchased from ThermoFisher Scientific (Supplementary Table S3). This was performed in a CFX384 Fast Real-Time PCR System (Bio-Rad Laboratories). Relative gene expression was calculated using the comparative method as described [40]. Briefly, *C*_t_ values were normalized to the housekeeping gene peptidylprolyl isomerase A (*Ppia*), which was unchanged between groups and has previously been shown to be a stable housekeeping reference gene [41]. 

### 2.14. High-Sensitivity Immunology Multiplex Assay

A Milliplex MAP Mouse High Sensitivity T Cell Panel (Millipore, Billerica, MA, USA) was performed on a Luminex 200 (Luminex, Austin, TX, USA) system using Xponent 3.1 software (Luminex), as per the manufacturer’s instructions. Quantification was performed by the Analytical Facility for Bioactive Molecules of the Centre for the Study of Complex Childhood Diseases, The Hospital for Sick Children, Toronto, Canada. 

### 2.15. Cytochrome c Oxidase (COX), Citrate Synthase (CS), and Short Chain β-Hydroxy-Acyl-CoA-Dehydrogenase (β-HAD) Enzyme Activity Assays

The maximal activities of citrate synthase (CS), cytochrome *c* oxidase (COX), and short chain β-hydroxy-acyl-CoA-dehydrogenase (β-HAD) were determined in homogenized SkM, as previously described [42]. All enzyme assays were performed utilizing a calibrated spectrophotometer (Cary Bio-300, Varion, Inc., Palo Alto, CA, USA). Detailed methods found in Supplementary Experimental Procedures. 

### 2.16. Histological Analysis

Briefly, tissues were immersed in 10% formalin with 90% PBS for 24 h and placed into 70% EtOH for paraffin embedding and subsequent hematoxylin & eosin (H&E) staining by the John Mayberry Histology facility at McMaster University Medical Centre. All imaging was undertaken on a Nikon 90*i* microscope under 10X magnification, and adipocyte size analysis was performed with the NIS Elements Analysis Software (Nikon, Inc., Melville, NY, USA).

### 2.17. Statistical Analysis 

Statistical analysis was performed by GraphPad Prism 7.00 (San Diego, CA, USA). To examine differences between groups, 1-way ANOVA was employed. If omnibus F-test(s) were significant, a Fisher’s least squares difference (LSD) post hoc test was used to determine group differences compared to only the HFD. For pre- and post-supplementation data (e.g., body weight, body fat percentage) a 2-way repeated measures ANOVA (group × time) with post-hoc used when appropriate. For respiration data, a 2-way ANOVA (titration x group) with post hoc was used where appropriate. Statistical significance was accepted at *p* < 0.05. All results are presented as means ± SEM.

## 3. Results

### 3.1. Supplementation with ME10 and/or ME7 Attenuated Accumulation of Fat Mass and Preserved Lean Body Mass, Which Was Further Enhanced When Combined with Exercise

**Figure 1:**
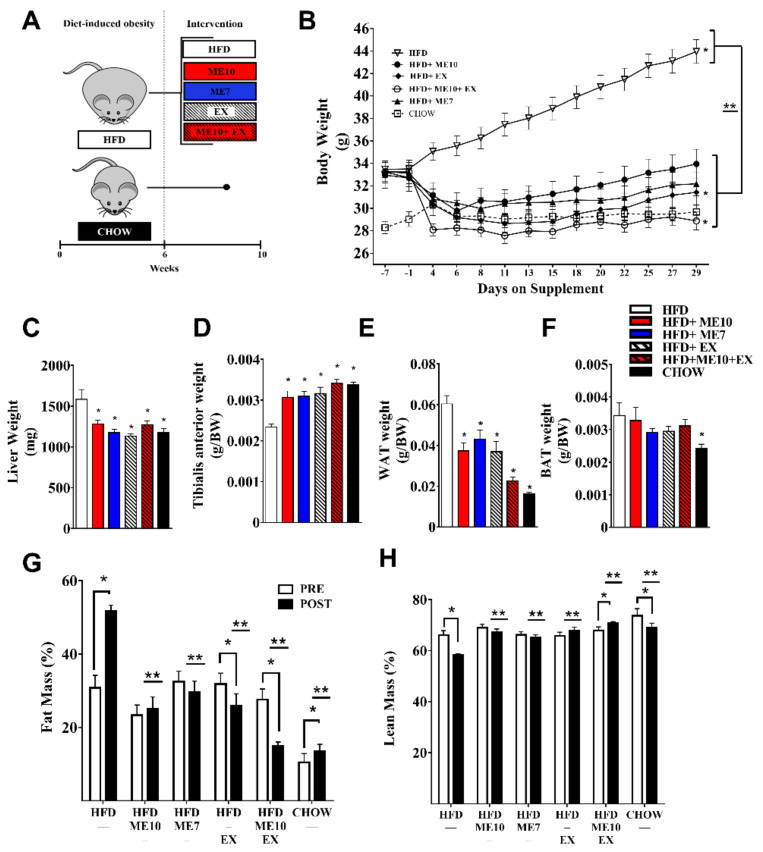
Metabolic enhancer (ME) supplementation and/or exercise stimulated weight loss despite the continuous provision of a high-fat diet. Experimental approach (**A**). Beginning at 6 weeks of age, C57BL6J mice consumed a high-fat diet (HFD) for 6 weeks. Animals subsequently remained on HFD but received ME supplementation and/or performed exercise for 4 weeks. Body weight (**B**) was measured throughout the experiment. Weights compared by 2-way RM ANOVA. *: *p* < 0.05 compared to Pre (Day -1) within groups, **: *p* < 0.05 compared to HFD following intervention. Relative tibialis anterior muscle weight (**C**), liver weight as a marker of hepatomegaly (**D**), relative intra-abdominal white adipose tissue (WAT) weight (**E**), and relative brown adipose tissue (BAT) weight recorded following animal sacrifice (**F**). 1-way ANOVA with Fisher’s LSD post-hoc test. *: *p* < 0.05 compared to the high-fat diet (HFD) group. Relative fat mass (**G**) and relative muscle mass (**H**) were recorded prior to (Pre, open bars) and following (Post, filled bars) using the Bruker minispec LF90II Body Composition Analyzer (NMR). Body composition compared by 2-way RM ANOVA. *: *p* < 0.05 compared with Pre within groups, **: *p* < 0.05 compared to HFD following intervention (filled bars). Data are represented as mean ± SEM.

By design, there were no significant differences in total body mass in the ME10, ME7, EX, or ME10 + EX groups compared to the HFD control mice prior to the intervention. After 4 weeks of intervention the HFD control animals showed a 31.2 ± 1.2% increase in BW (*p* < 0.05, Figure 1B). BW was unchanged in the ME10 (+3.9 ± 2.1%, *p* > 0.05) and ME7 (−3.0 ± 1.1%, *p* > 0.05) groups; both groups were significantly lower than those of the HFD group (*p* < 0.05, Figure 1B) following the intervention. The EX (−4.0 ± 2.4%) and ME10 + EX (−12.7 ± 2.1%) groups had a significant reduction in BW; both groups had significantly lower BW as compared to the HFD group (*p* < 0.05, Figure 1B). The sedentary CHOW group did not change significantly in weight (+2.2 ± 0.7%, Figure 1B) during the experiment. 

We examined whether the marked weight loss following ME supplementation was global or tissue specific. HFD control animals had a greater liver weight, consistent with hepatomegaly (*p* < 0.05, Figure 1C). Both with and in the absence of exercise, animals given ME10 and ME7 had larger relative TA weights compared to HFD, respectively (*p* < 0.05, Figure 1D). Conversely, all animals on experimental diets and/or performing exercise interventions had a lower relative WAT weight (*p* < 0.05, Figure 1E). There were no differences in relative BAT weight between the HFD and supplementation diet/intervention groups, but CHOW animals did have a lower BAT depot size (*p* < 0.05, Figure 1F). Fat and muscle organ weights confirmed findings in vivo via NMR. We observed significant increases in FM (%) in the HFD control group, whereas animals supplemented with ME7 and ME10 were unchanged. In addition, the EX and ME10 + EX groups showed a significant reduction following intervention (*p* < 0.05, Figure 1G). Following the intervention, all groups had a significantly lower body fat percentage than the HFD group. The HFD and sedentary CHOW animals had a significant decrease in relative lean mass (LM) over the intervention period. Lean mass was unchanged with ME7 and ME10 but tended to increase in EX (*p* = 0.07) and increased in combinatory ME10 + EX (*p* < 0.05, Figure 1H). All groups had a significantly higher relative lean mass as compared to the HFD animals following the intervention. 

### 3.2. Weight Loss Not Exclusively Dependent on Appetite Suppresion

Throughout the intervention period, relative food intake (g/BW/day) was significantly decreased in HFD animals but increased in the experimental diet groups during the invention (*p* < 0.05, Figure S2A). Absolute food intake (g/day) was lower in ME7 and ME10 animals (*p* < 0.05, Figure S2B) compared to HFD, but remained unchanged in the groups in which the greatest mean weight loss occurred (EX, ME10 + EX, *p* > 0.05). 

### 3.3. Deleterious Impact of HFD-Induced Weight Gain on Exercise Ameliorated with Supplementation

To evaluate the effects of ME7 and ME10 supplementation independently or in conjunction with exercise training (ME10 + EX) upon aerobic capacity, an exercise capacity test was performed. Following the intervention, the HFD control and ME10 group exhibited significant decreases in maximal running capacity, whereas the ME7, EX, and ME10 groups did not (*p* < 0.05, Figure S2C). 

### 3.4. Exercise and Supplementation Upregulated Mitochondrial Biogenesis and mRNA Abundance of Markers of the Browning Program in WAT

**Figure 2:**
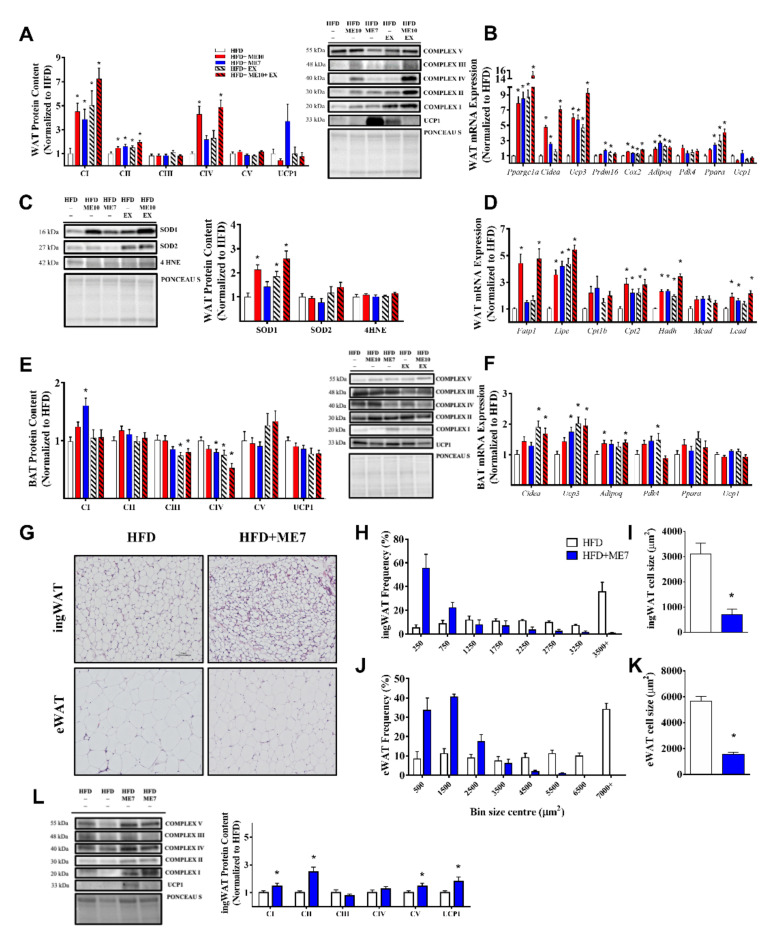
Metabolic enhancer (ME) supplementation and/or exercise induces greater OXPHOS protein expression, mRNA expression of markers of the browning program, and mitochondrial biogenesis during HFD in epididymal white adipose tissue (eWAT). Protein expression (**A**) of OXPHOS complexes (complex I–V) and uncoupling protein 1 (UCP1) in WAT tissue, expressed relative to the HFD group with representative blots. mRNA expression (**B**) of browning-related genes in WAT. Protein expression (**C**) of antioxidant SOD1 and SOD2, and marker of lipid peroxidation 4-Hydroxynonenal (4-HNE) with representative blots. Gene expression (**D**) indicators of lipid transportation and β-oxidation in WAT. Protein expression (**E**) of OXPHOS complexes (complex I–V) and uncoupling protein 1 (UCP1) in in brown adipose tissue (BAT). mRNA expression (**F**) of browning-related genes in BAT. 1-way ANOVA with Fisher’s LSD post-hoc test. *: *p* < 0.05 compared to high-fat diet (HFD) group. Data are represented as the mean ± SEM (*n* = 10–12 per group). To confirm observations in eWAT, we performed an experiment in an additional subset of animals, examining inguinal white adipose tissue (ingWAT). (**G**) Representative haematoxylin and eosin stain of ingWAT and eWAT depots (magnification X 10) in HFD and HFD + ME7 groups. (**H**) Frequency distribution of ingWAT cell size and (**I**) mean cell size of ingWAT. (**J**) Frequency distribution of eWAT cell size and (**K**) mean cell size of eWAT. Protein content (**L**) of OXPHOS complexes and UCP1 in ingWAT tissue, expressed relative to the HFD group with representative blots. Student’s *t* test, *: *p* < 0.05 compared to the high-fat diet (HFD) group. Data are represented as mean ± SEM (*n* = 6 per group).

Mitochondria OXPHOS-related protein content in eWAT improved in a complex-specific manner in response to supplementation and/or exercise. Compared to HFD, supplemented and/or exercising animals had greater complex I, II, and IV content (*p* < 0.05, Figure 2A). The final common pathway indicating browning/‘beiging’ is the induction of UCP1 protein expression and mitochondrial biogenesis [15]. UCP1 protein was detectable in eWAT, although considerable variation was observed within groups. The ME7 group exhibited the greatest mean expression compared to the HFD group (*p* > 0.05, Figure 2A). We observed a similar pattern in *Ucp1* mRNA expression (*p* > 0.05, Figure 2B), as well as greater mRNA expression of markers of the browning program in eWAT, including *Cidea*, *Prdm16*, *Pppara*, and *Adipoq,* in experimental diet compositions (ME7, ME10) and exercise groups (*p* < 0.05, Figure 2B).

### 3.5. Supplementation and Exercise Increased SOD1 Antioxidant Defense in WAT

Oxidative stress may play a role in the initiation or exacerbation of obesity [10,43]. Exercise has been shown to have antioxidant effects in muscle [44,45]. Therefore, we examined whether supplementation and/or chronic exercise facilitates antioxidant defense during a continued HFD challenge. We found a significantly greater SOD1 protein content in the ME10, EX, and combinatory groups relative to HFD (*p* < 0.05, Figure 2C), with no observable differences in SOD2 (Figure 2C) or lipid peroxidation products (4-HNE) (Figure 2C). 

### 3.6. Exercise and Supplementation Improved WAT Lipid Transport and Metabolism mRNA Expression

Adipose tissue plays an important role in the regulation of lipid availability [46], and thus we examined whether markers of lipid transport and β-oxidation were improved with supplementation. We observed higher mRNA expression of markers responsible for cellular (*Fatp1*) and mitochondrial (*Cpt2*) uptake of fatty acids in experimental groups compared to HFD (*p* < 0.05, Figure 2E), with non-significant but similar trends in cytosolic membrane transport (*Cpt1b*). This dysregulation was mirrored by mRNA expression of enzymes linked to mitochondrial fatty acid β-oxidation (*Hadh, Lcad*) (*p* < 0.05, Figure 2D). 

### 3.7. OXPHOS Protein Expression Was Altered in a Complex-Specific Manner in BAT Following Exercise and Supplementation

Brown adipose tissue (BAT) is important for thermogenesis and energy homeostasis, has a high mitochondrial content, and plays a role in the development of diet-induced obesity [47]. Paradoxically, the BAT response to exercise has shown counter-intuitive effects, with a reduction in BAT mass and UCP1 content [48]. We observed that in the HFD + ME7 group, there was significantly higher complex I expression as compared with the HFD control group (*p* < 0.05, Figure 2E). However, there was a significantly lower expression in complex IV protein in ME7 (*p* < 0.05) as well as complex III and IV in the EX and ME10 + EX groups as compared to the HFD group in BAT (*p* < 0.05, Figure 2E). There was no significant difference in UCP1 expression between experimental groups (*p* > 0.05). We observed higher mRNA abundance related to mitochondrial function, lipid fusion, ROS defense, and energy homeostasis in BAT primarily in the EX and ME10 + EX groups (*p* < 0.05, Figure 2F).

### 3.8. ME7 Supplementation Improved OXHOS Protein Expression in Inguinal White Adipose Tissue Depots in a Similar Manner to Epidydimal WAT

To confirm the influence of HFD + ME7 supplementation on mitochondrial OXPHOS protein expression in epidydimal WAT, we followed up with an examination of inguinal white adipose tissue (ingWAT) in a subset of animals (HFD, *n* = 6; HFD + ME7, *n* = 6). These findings showed a consistent and significant reduction in adipocyte size for ingWAT (*p* < 0.05, Figure 2H,I) as well as eWAT (*p* < 0.05, Figure 2J,K) in the ME7 group as compared to the HFD control group. Confirming our previous observations in the epidydimal WAT depot, OXHPHOS complexes I, II, and V were significantly greater in ME7 compared to HFD (*p* < 0.05, Figure 2L). As in eWAT, UCP1 protein was detectable in ingWAT and mean expression was higher in ME7 (*p* < 0.05, Figure 2L).

### 3.9. Exercise and Supplementation with ME7 and ME10 Limits Inflammation in WAT and Systemic Circulation

**Figure 3:**
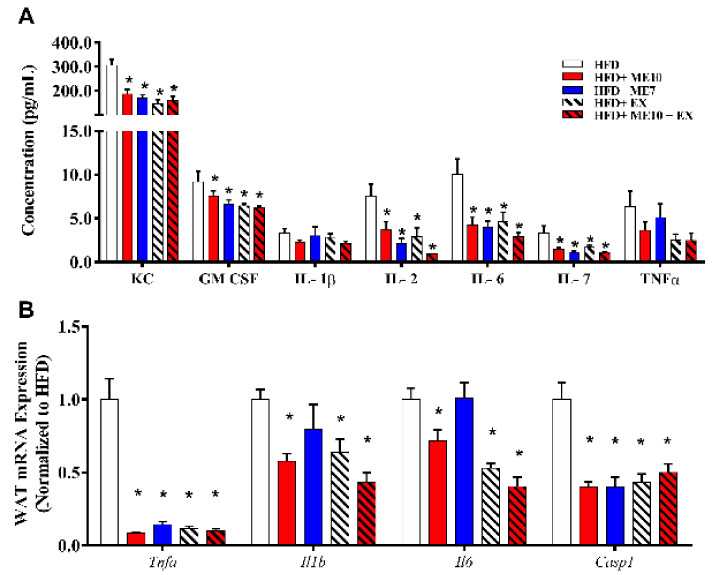
Metabolic enhancer (ME) supplementation and/or exercise reduces circulating inflammatory cytokines (**A**), and simultaneously reduces mRNA expression (**B**) in white adipose tissue (WAT). 1-way ANOVA with Fisher’s LSD post-hoc test. *: *p* < 0.05 compared to the high-fat diet (HFD) group. Data are represented as the mean ± SEM (*n* = 8–12 per group).

The release of inflammatory cytokines from adipocytes [49,50] can cause the recruitment of immune cells to growing WAT, which propagates local and systemic inflammation [51]. Therefore, we examined supplementation and/or exercise on circulating markers of inflammation via a multiplex immune-bead assay. IL-8 (KC), GM-CSF, IL-2, IL-6, IL-7 concentrations were lower in all groups compared to the HFD animals (*p* < 0.05, Figure 3A), with non-significant but similar trends in IL-1β and TNF-α (*p* > 0.05). To confirm whether the decrease in systemic cytokines reflected an altered expression in adipose tissue, we measured markers of inflammation in eWAT. Similarly, there was lower mRNA expression in *Tnfa*, *Il1b*, *Il6*, and *Casp1* in animals undergoing supplementation and/or exercise (*p* < 0.05, Figure 3B). 

### 3.10. Exercise and Supplementation Ameliorated the Alteration of Mitochondrial Function in SkM Compared to HFD

**Figure 4:**
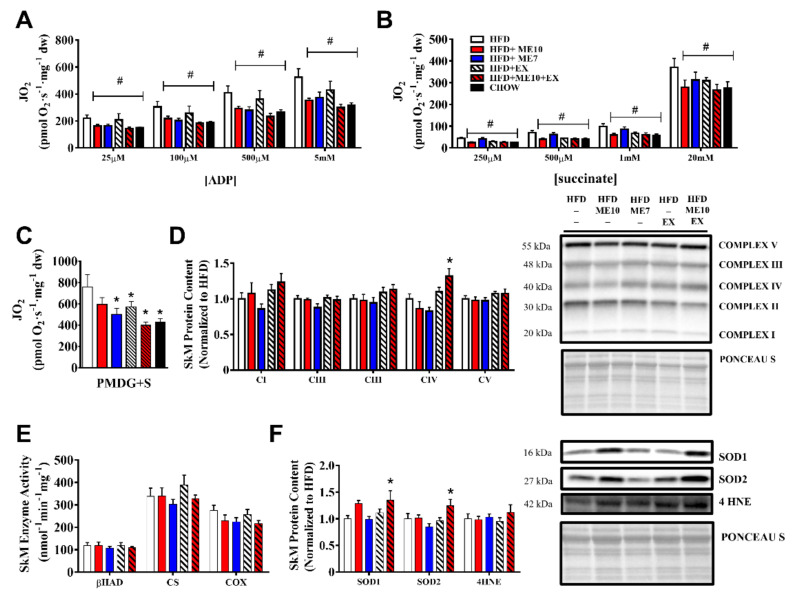
Alteration of mitochondrial bioenergetics and protein content following metabolic enhancer (ME) supplementation and/or exercise in skeletal muscle. Complex I-supported ADP-stimulated respiratory kinetics (**A**) were examined via the titration of submaximal ADP (25, 100, and 500 µM) and maximal ADP (5 mM) in the presence of 5 mM pyruvate and 2 mM malate. #: main effect across all [ADP] titrations, HFD compared to all other experimental groups (*p* < 0.05). Complex II-supported respiration (**B**) was determined through the stepwise addition of succinate (FADH_2_) in the presence of 5 mM ADP and 10 µM rotenone (to inhibit and prevent superoxide generation at complex I). Titration experiments examined via 2-way ANOVA. (**C**) Maximal ADP-stimulated respiration in the presence of complex I- and II-linked substrates, PMDG + S, pyruvate (P), malate (M), ADP (D), glutamate (G) + succinate (S). 1-way ANOVA with Fisher’s LSD post-hoc test. *: *p* < 0.05 compared to the high-fat diet (HFD) group. Protein content (**D**) of OXPHOS complexes (Complex I–V) in SkM tissue, expressed relative to the HFD group with representative blots. 1-way ANOVA with Fisher’s LSD post-hoc test. *: *p* < 0.05 compared to the high-fat diet (HFD) group. Maximal enzyme activity of (**E**) β-HAD, COX, and CS in skeletal muscle (*p* > 0.05). Protein content (**F**) of antioxidant SOD1 and SOD2, and marker of lipid peroxidation 4-Hydroxynonenal (4-HNE) with representative blots. 1-way ANOVA with Fisher’s LSD post-hoc test. *: *p* < 0.05 compared to the high-fat diet (HFD) group. Data are represented as the mean ± SEM (*n* = 8–12 per group).

To examine skeletal muscle mitochondrial function, we utilized high-resolution respirometry and complex-specific assays. ADP-stimulated respiration was greater in the SkM of HFD (*p* < 0.05, Figure 4A) compared to all other groups (main effect for group). Notably, animals in all experimental groups demonstrated ADP-stimulated oxygen consumption rates that were similar in nature to those of the CHOW-fed animals (i.e., suggesting a normalization of mitochondrial function). SkM complex II-supported respiration (succinate-induced FADH_2_ synthesis) was significantly higher in the HFD group compared to all experimental groups (*p* < 0.05, Figure 4B) across physiological, submaximal, and maximal concentrations of succinate (main effect for group). SkM Complex I + II state 3-supported respiration (PMDG + S) was significantly higher in HFD animals compared to all experimental groups (*p* < 0.05, Figure 4C), with the exception of ME10 (*p* = 0.08).

### 3.11. Improvement in SkM Mitochondrial Respiration Independent of Mitochondrial Protein Expression Following Supplementation

Electron transport chain protein content measured in the quadriceps was largely unchanged in the experimental groups. Despite a relatively short exercise period, there was a higher abundance of complex IV protein in the ME10 + EX group as compared to HFD animals (*p* < 0.05, Figure 4D). These findings suggest the differences in observed via high-resolution respirometry were not due to changes in mitochondrial content. There was no significant intervention effect on SkM β-HAD maximal activity (a marker of β-oxidation capacity), CS maximal activity, mitochondrial COX maximal activity (*p* > 0.05, Figure 4E), or the ratio of COX/CS (the COX activity relative to total mitochondria; data not shown *p* > 0.05). 

### 3.12. Supplementation and Exercise Increased SOD1 Antioxidant Defense in SkM

ME10 + EX animals had increased SOD1 SkM protein content (*p* < 0.05, Figure 4F), while ME10 animals showed a trend toward an increase in SOD1 protein content (*p* = 0.06, Figure 4F). ME10 + EX mice showed a significantly higher SOD2 content (*p* < 0.05, Figure 4F). Lipid peroxidation-derived 4-HNE did not significantly differ between experimental interventions (*p* > 0.05, Figure 4F).

### 3.13. In Vivo Lipid Oxidation Rates, Animal Activity, and Tissue-Specific AMPK Phosphorylation Increased Following a Short-Term (3d) Supplementation Period 

**Figure 5:**
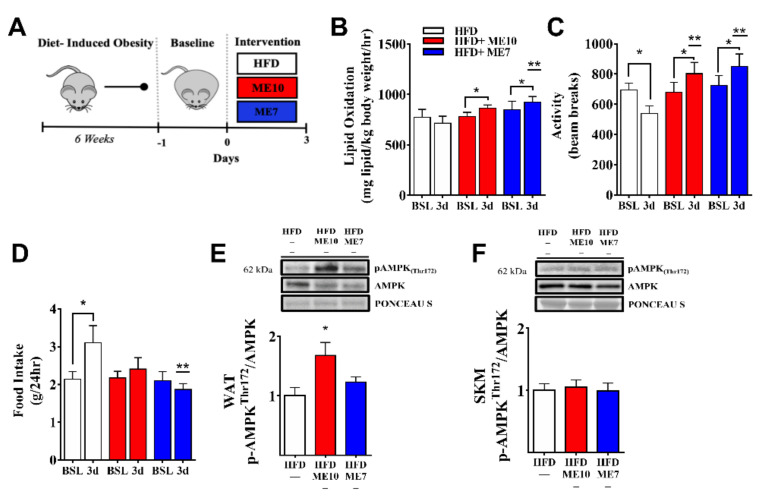
Short-term supplementation (3 days) of metabolic enhancer (ME) facilitates metabolic activity assessed via CLAMS. Experimental approach (**A**). Supplementation for a 3-day period (3d) increases lipid oxidation (**B**) and animal activity (**C**), and alters food intake compared to baseline (BSL) (**D**). *: *p* < 0.05 compared with BSL within groups, **: *p* < 0.05 compared to HFD following intervention (3d). Short-term supplementation increases AMPK phosphorylation (Thr172) in white adipose tissue (WAT) (**E**) but not skeletal muscle (SkM) (**F**). Representative blots of the total AMPK and *p*-AMPK_Thr172_ in adipose tissue (WAT) and skeletal muscle (SkM), respectively. *: *p* < 0.05 compared to the high-fat diet (HFD) group. Data are represented as the mean ± SEM (*n* = 7–8 per group).

In both 4-week supplementation experimental approaches (Approach 1; Figure S1, and Approach 2; Figure 1), acute weight loss occurred at as early as after the first 3 days of supplementation. Therefore, we examined lipid oxidation rates and activity via CLAMS following a 3-day period (Figure 5A). Lipid oxidation was unchanged in the HFD group but increased in the ME10 and ME7 groups (*p* < 0.05, Figure 5B), such that values for the ME7 group were significantly higher than those of HFD animals (*p* < 0.05). Activity levels of ME7 and ME10 mice increased after 3 days of supplementation, whereas the HFD group showed significantly reduced activity (*p* < 0.05, Figure 5C). Food consumption (g/24 h) during the experimental period was increased in the HFD group but was unchanged in the ME10 and ME7 groups (*p* < 0.05, Figure 5D). AMPK signaling is indirectly activated by catecholamine secretion in response to β-adrenergic stimulation [52] and has previously been shown to be upregulated in response to some ME components such as GTE [53]; therefore, immunoblots for *p*-AMPK_Thr172_ were performed. ME10 supplementation increased AMPK phosphorylation (*p* < 0.05, Figure 5E) in eWAT. There were no significant differences detected in SkM (*p* > 0.05, Figure 5F).

## 4. Discussion

The current study has demonstrated that two multi-ingredient oral supplements (ME7 and ME10), developed to target key aspects involved in the pathogenesis of obesity, significantly reduced WAT content (i.e., obesity) in HFD mice. HFD animals gained weight and relative FM, developed hepatomegaly, and increased their WAT content. ME7 and ME10 supplementation ameliorated weight gain, despite the caloric surplus of a ~60% HFD, without exercise. When consumed in combination with exercise, ME10 + EX animals presented extensive relative FM and BW reductions, such that animals were similar to CHOW animals. ME7 and ME10 drove a greater basal physical activity rate and basal oxidation rate in vivo. Importantly, many of the effects of ME supplementation were similar to the exercise intervention (EX). In contrast to our *a priori* hypothesis, combining supplementation and exercise did not negate the benefits of exercise training. Molecular adaptations related to the dietary supplementation were observed in WAT (e.g., higher mitochondrial content, indices of browning, lower inflammation), with the amelioration of HFD-induced alterations in mitochondrial respiration observed in SkM. 

One of the most sought-out effects is the phenotypic shift from WAT (energy storing) to beige adipose tissue (more metabolically active). Some WAT depots can convert to a ‘brown-like’ or ‘beige’ state, characterized by mitochondrial biogenesis and an increase in UCP1^+^, multilocular adipocytes [54]. Therefore, ‘beiging’ or browning of WAT through exercise and/or pharmacological activation represents an important target for the development anti-obesity strategies. We observed greater mRNA expression for markers of beige adipocytes including *Cidea*, *Ppara*, *Pdk4*, and *Prdm16* [55] in WAT following the intervention, concomitant with an increase in OXPHOS protein expression. The provision of ME supplementation led to an increase in UCP3 mRNA expression (shown to be correlated with UCP1 expression [56,57]) and may also play a role in reducing ROS [58]. Taken together, these transcription factors are critical for oxidative metabolism and mitochondrial biogenesis in WAT [59,60]. Examination of eWAT and ingWAT depots confirmed a significant shift in adipocyte morphology following ME7 supplementation as compared to HFD. mRNA expression of browning and mitochondria-related genes were also increased in the exercising groups, in keeping with the notion that exercise can promote this adaptation in adipose tissue [61,62]. While the ‘beiging’ process in response to exercise likely occurs via different molecular pathways [63] to those of ME supplementation, our data would suggest that supplementation can facilitate a shift in WAT phenotype in a similar fashion. Interestingly, we observed altered UCP1 protein expression in WAT predominately following supplementation in ME7, despite higher mRNA expression of browning-related markers across other intervention groups. While the UCP1-dependent browning process is certainly more studied, growing evidence would suggest UCP1-independent mechanisms as well [64,65,66,67]. Indeed, adipose tissue thermogenesis can be driven by futile creatine cycling [66,68], and so future work should address how ME with (ME10) or without (ME7) creatine supplementation facilitates the observed aspects of WAT browning. Interventions such as ME supplementation and EX may elicit different effects on classical BAT, known for densely packed mitochondria and thermogenic capacity [69]. In line with previous studies examining exercise, we found a significantly lower BAT mass compared to HFD [70,71], with similar observations following ME supplementation. There is conflicting evidence regarding the expression of UCP1 in response to both HFD [72] and EX [48,73] in BAT. We observed a significant shift in the expression of OXPHOS proteins in response to experimental interventions (ME and/or EX), concomitant with increased mRNA expression of genes related to mitochondrial function [74], ROS defense [58] and energy homeostasis [75] in BAT, without a significant change in UCP1 expression. Recent work [76] demonstrated that an imbalance in the ETC proteome within the BAT can regulate systemic metabolism at room temperature and ultimately lead to an improved metabolic phenotype. Together, our findings suggest the ME supplementation and/or exercise altered BAT ETC protein expression during HFD, despite a smaller BAT tissue size. 

Brown adipogenesis and/or brown adipocyte differentiation in vitro [77] occurs in response to AMPK activation. Pharmacological activation of AMPK has been shown to increase WAT-specific UCP1 expression [78]. AMPK activation is known to promote fatty acid oxidation [79], while adipose tissue-specific deletion/ablation of both AMPK β1 and β2 subunits compromises WAT lipid metabolism [36,78]. Compared to HFD, multi-nutrient supplementation resulted in increased AMPK activation in WAT. Our findings are consistent with the literature suggesting that AMPK activation in WAT occurs concomitantly with browning and increased energy expenditure [80]. Norepinephrine-mediated upregulation of PGC1-α and mitochondrial protein expression has been shown to be regulated by AMPK in eWAT in mice [81]. In addition, exercise has also been shown as a potent stimulator of AMPK in adipose tissue [82] and is a known stimulator of PGC-1α. In line with this, we found greater total OXPHOS protein expression following the intervention, underscoring the increase in mitochondrial content in eWAT. PGC1-α mRNA expression was upregulated even after 30 days, suggesting that the stimulus for mitochondrial biogenesis was still present. 

Herein, the primary driver of weight management in both the absence (HFD + ME7, HFD + ME10) or presence (HFD + EX, HFD + ME10 + EX) of exercise is challenging to isolate given the multi-ingredient nature of the supplement and the systemic benefits of exercise. 

The active components of GTE include epigallocatechin-3-gallate (EGCG), the most abundant catechin of green tea in terms of content [83] and bioactivity [84]. The activation of AMPK by EGCG has been shown both in vivo [85] and in vitro [86], and green tea catechins can increase mRNA levels of enzymes for fatty acid β-oxidation [86]. EGCG has been shown to inhibit catechol O-methyltransferase, the enzyme that degrades norepinephrine, thereby extending its presence and availability in the synaptic cleft [87]. Caffeine, occurring naturally in GTE, GBE, and BTE also influences the activity of the sympathetic nervous system (SNS) through the inhibition of phosphodiesterase [88], an enzyme which degrades intracellular cyclic AMP (cAMP). The prolonged presence of cAMP facilitates lipolysis, increasing fatty acid availability for fuel use [89]. However, the effects of caffeine at physiological concentrations (like the low dosages provided herein) in vivo are instead likely due to adenosine receptor antagonism [87]. Further, caffeine may also stimulate a reduction in food consumption in both mouse [90] and human [91] models. However, there are also reports indicating caffeine can also increase food consumption in various mouse models [92,93]. Here, we found that animals in the ME7 and ME10 groups had reduced food consumption across the experimental period which may, in part, facilitate the observed mitigation of weight gain in these groups. Given that mice provided HFD diet *ad libitum* tend to overconsume food [94,95,96] and have altered meal patterns [97], we speculate that the provision of different aspects within the experimental ME7 and ME10 diets may help to reduce this phenomenon. Indeed, animals consuming the HFD + ME10 and HFD + ME7 diets consumed the same caloric intake as animals on a standard chow diet across the experimental period. Interestingly, we found that the EX and ME10 + EX group, which showed significant reductions in weight (decrease of ~4% and ~13%, respectively) consumed the same amount of food (g/day) as the HFD group, which exhibited a significant weight gain (increase ~31%). While appreciating food consumption and energy metabolism in the murine model is complex and challenging [98,99,100], our observations underscore the notion that metabolic activation through activity (EX) or nutraceutical-enhanced activity (ME10 + EX) is able to facilitate weight loss despite the ongoing insult of HFD. Further, decaffeinated GTE can also activate AMPK [101] and given our results, it is likely that caffeine is not the main driver of weight loss. Forskolin (derived from *coleus forskholi*) has been shown to stimulate lipid mobilization and oxidation through direct stimulation of adenylate cyclase, raising cAMP levels and upregulating HSL [102]. HSL is integral for the release of free fatty acids from storage within adipose tissues, with decreased HSL resulting in impaired lipolysis in WAT [103]. Our findings would support that ME supplementation and/or exercise may facilitate the liberation, transport, and ultimate breakdown of free fatty acids, given the increased expression of fatty acid transporters and markers of β-oxidation in WAT. Together, the reported obesity-related impairment of fatty acid oxidation [104] may be alleviated with exercise and/or supplement therapy. Critically, we observed that the phenotypic reduction in fat mass was the greatest in dietary supplementation that combined ingredients (ME7, ME10) known to improve mitochondrial function as well as ingredients known to promote weight loss. The synergy in these specific ingredients is highlighted by extensive fat loss in the HFD + ME7 and HFD + ME10 groups. This notion is further underscored based on the less robust fat loss observed in animals consuming proposed weight loss-inducing agents (i.e., the WL5 group) or only mitochondrial-enhancing ingredients (i.e., Mito5 group) during Experimental Approach 1 (Figure S1). Together, these results suggest that the combinatory approach using mitochondrial and fat loss-targeting ingredients is uniquely beneficial.

Adipose tissue is a functional, secretory organ capable of producing adipokines [105]. Nutritional supplementation with ME7, ME10- and/or exercise training was able to facilitate a lowered circulating concentration of cytokines which have been associated with obesity (including IL-6 [106,107], IL-7 [108], and IL-8 [109]), which was mirrored by lowered mRNA expression of these markers in WAT. These findings are in line with previous literature regarding components of the multi-ingredient supplement. The treatment of *ob*/*ob* mice with CoQ10, an effective lipid-soluble mitochondria-specific antioxidant and electron acceptor during respiration, resulted in a reduction in mRNA TNF-α expression [110]. Vitamin E, with similar antioxidant properties as CoQ10, has been shown to lower IL-6 in eWAT of HFD-fed mice [111] and also appears to work synergistically with α-LA, another known antioxidant known to improve the obese phenotype [25]. Together, this suggests that multi-nutrient supplementation containing these ingredients would have an additive and/or synergistic benefit. Exercise training can reduce chronic inflammation, particularly in an obese phenotype with elevated levels of inflammatory biomarkers [112], possibly by reducing inflammation at the level of adipose tissue by reducing macrophage infiltration [113]. 

Mitochondrial dysfunction has also been reported in skeletal muscle with obesity or metabolic diseases such as diabetes [104,114]. Interestingly, we observed that the HFD group improved respiration in SkM, as assessed by sensitivity to ADP and succinate (for complex II specific respiration). These findings suggest that mitochondrial respiration is increased through compensatory mechanisms independent of mitochondrial content, in line with some previous findings regarding HFD [115]. Mitochondrial adaptations with HFD are inconsistent, with some reporting no reductions in oxidative capacity [116,117] and others reporting an increase in mitochondrial content and/or mitochondrial respiration despite the development of insulin resistance [118]. The process may be dynamic, with short-term exposure to HFD increasing mitochondrial respiration, and long-term exposure resulting in impairment [119]. In line with this, our findings suggest the ‘insult’ of the HFD may temporarily enhance respiration (through mechanisms such as increased ADP sensitivity), and prolonged or chronic HFD may lead to a loss of homeostatic balance. Indeed, in adipose tissue, deleterious effects of HFD (e.g., insulin resistance) may be driven by the mitochondrial emission of ROS [120,121] or from dysfunction of succinate dehydrogenase (SDH) in WAT [122], and thus a limitation of this study is that we exclusively examined SkM and not BAT and/or WAT through the lens of high-resolution respirometry. Importantly, the observation that ME7 and ME10 supplementation ameliorated HFD-induced alterations in SkM mitochondrial respiration suggests a mitigation of the signal cascade that may drive the compensatory hormesis observed in the SkM of the HFD group. 

ME7 and ME10 supplementation acts to lower inflammation, upregulate crucial fatty acid transporters, and promote the activation of a ‘beiging’ of white adipose tissue at the protein and/or mRNA expression level—possibly through the activation of AMPK. In vivo, we found that the supplementation is related to higher basal oxidation rates and an increased basal physical activity level, likely promoting energy expenditure. Future trials in humans are required to confirm if combinatory supplementation based on these results in the murine model indeed represents a viable anti-obesogenic strategy.

## Supplementary Materials

The following are available online at https://www.mdpi.com/article/10.3390/nu13113726/s1. Supplementary Figure S1: Preliminary experiment performed to optimize multi-ingredient supplementation (Experimental Approach 1). Figure S2: Experimental period food consumption and exercise performance. Table S1: Experimental diet composition. Table S2: Antibody information. Table S3: Taqman probe information. Supplementary experimental procedures.
