# Supplementary material for: A Novel Multi-Ingredient Supplement Activates a Browning Program in White Adipose Tissue and Mitigates Weight Gain in High-Fat Diet-Fed Mice
Source: Nutrients. 2021 Oct 22;13(11):3726. doi: 10.3390/nu13113726 (PMC8623014; doi:10.3390/nu13113726)
Supplement: Supplementary file 1 [file nutrients-13-03726-s001.zip › nutrients-1219802-supplementary.pdf]

SUPPLEMENTARY MATERIALS

TITLE: A Novel Multi-Ingredient Supplement Activates a Browning Program in White Adipose Tissue and Mitigates Weight Gain in High-Fat Diet-Fed Mice

AUTHORS: Joshua P Nederveen[1], Katherine Manta[1], Adam L Bujak[2], Alexander C Simone[1], Matthew R Fuda[1], Mats I Nilsson[2], Bart P Hettinga[2], Meghan C Hughes[3], Christopher GR Perry[3], Mark A Tarnopolsky[1,2].

INSTITUTIONS:

[1]Department of Pediatrics, Faculty of Health Sciences, McMaster University Medical Center (MUMC), 1200 Main St. W, Hamilton, ON L8N 3Z5, Canada

[2]Exerkine Corporation, MUMC, 1200 Main St. W, Hamilton, ON L8N 3Z5, Canada

[3]Muscle Health Research Centre (MHRC), School of Kinesiology & Health Science, York University, 4700 Keele Street, Toronto, ON M3J 1P3, Canada

*Corresponding author
E-mail: tarnopol@mcmaster.ca

Supplementary Table S1. Experimental Diet Composition

Experimental Diet Name	% kcal from fat	Calories (kcal/g)	Supplementation (by weight)
HFD[1,2,3]	60.3	5	No supplementation provided
HFD[1,2,3] ME10	62.5	5	0.25% GTE, 0.13% BTE, 0.25% GCBE 0.25% CLA, 0.005% FSK, 1% BE, 0.25% CoQ10; 0.1% ALA, 1% Cre 1000IU/kg of VitE
HFD ME7[2,3]	61.7	5	0.375% GTE, 0.25% GCBE, 0.005% FSK 1% BE, 0.25% CoQ10, 0.1% ALA 1000IU/kg of VitE
HFD WL5[1]	60.8	5	0.25% GTE, 0.13% BTE, 0.25% GCBE 0.25% CLA, 0.005% FSK
HFD Mito5[1]	61.9	5	1% BE, 0.25% CoQ10; 0.1% ALA, 1% Cre, 1000IU/kg of VitE
CHOW[1,2]	17.1	3	No supplementation provided

Supplementary Table S1. HFD; high fat diet, GTE; green tea extract, BTE; black tea extract, GCBE; green coffee bean extract, CLA; conjugated linoleic acid, FSK; forskolin, BE; beetroot extract, CoQ10; Coenzyme Q10 (ubiquinone), ALA; alpha (α) lipoic acid, Cre; creatine, VitE; vitamin E. All custom diets were composed by Teklad (Envigo). Chow diet is the standard Teklad 8640. Abbreviations for the Experimental diets: ME (metabolic enhancer), WL (weight loss), Mito (mitochondrial enhancer). ME7 and ME10 denote the number of ingredients, respectively. [1]; diet used in preliminary (Study 1), [2]; diet used in main study (Study 2), and [3]; diet used in short-term feeding study (3d; Study 3).

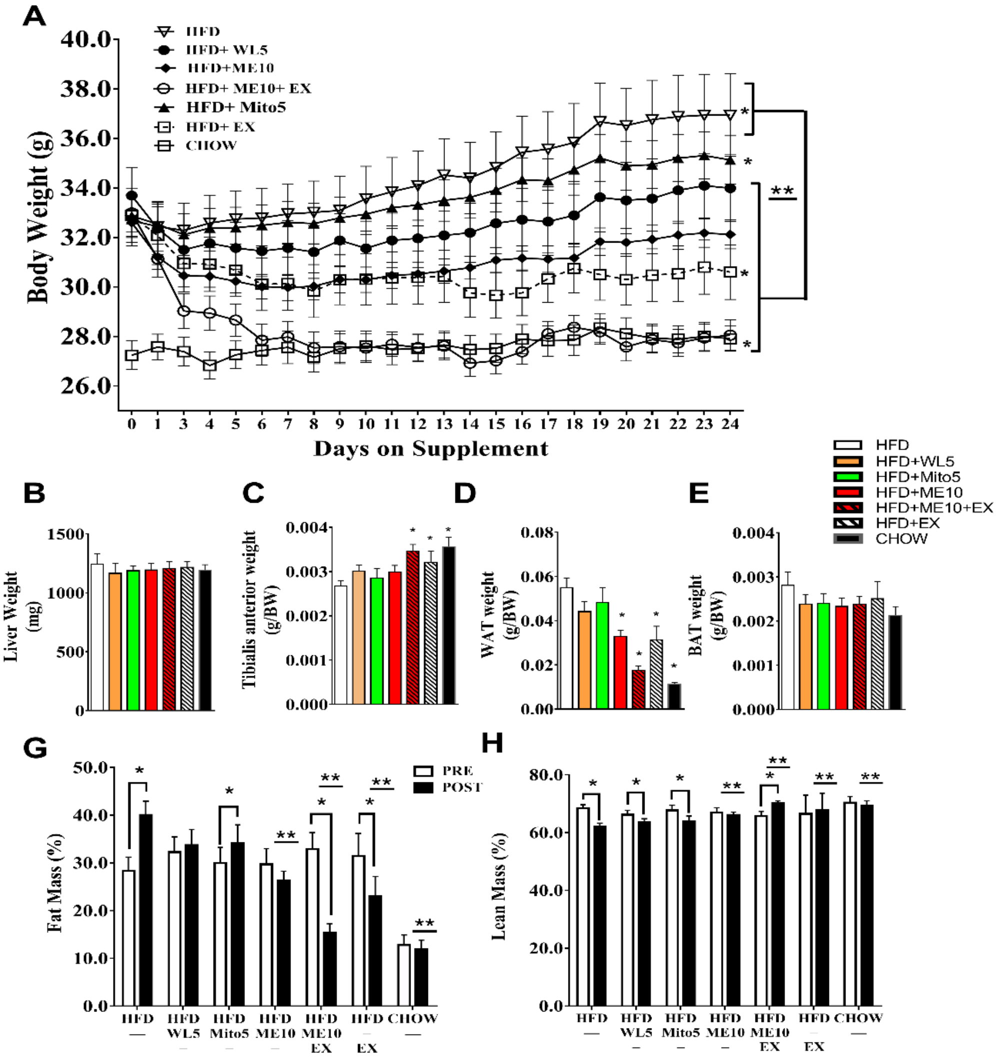

Figure S1. Preliminary experiment performed to optimize multi-ingredient supplementation (Experimental Approach 1). Various multi-ingredient compositions were examined. Weight loss ingredients (WL5), mitochondria enhancing ingredients (Mito5) and combined metabolic enhancer (ME10) supplementation and/or exercise were examined, refer to Supplementary Table 1 for composition. Beginning at 6 weeks of age, C57BL6J mice consumed high fat diet (HFD) for 6 weeks. Animals subsequently remained on HFD but consumed ME supplementation and/or performed exercise for ~4 weeks. Body weight (**A**) was measured throughout the experiment. Weights compared by two-way RM ANOVA. *; $p<0.05$ compared to Pre (Day -1) within group, **; $p<0.05$ compared to HFD following intervention. Gross liver weight as a marker of hepatomegaly (**B**), relative *tibialis anterior* muscle weight (**C**), relative intra-abdominal white adipose tissue (WAT) weight (**D**), and relative brown adipose tissue (BAT) weight (**F**) being recorded following animal sacrifice. Tissue weights compared by one-way ANOVA. *; $p<0.05$ compared to high-fat diet (HFD) group. Relative fat mass (**G**) and relative muscle mass (**H**) were recorded prior to (Pre; open bars) and following (Post, filled bars) via Bruker minispec LF90II Body Composition Analyzer. Body composition compared by two-way ANOVA. *; $p<0.05$ compared with Pre within group, **; $p<0.05$ compared to HFD following intervention (filled bars). Data are represented as mean ± SEM.

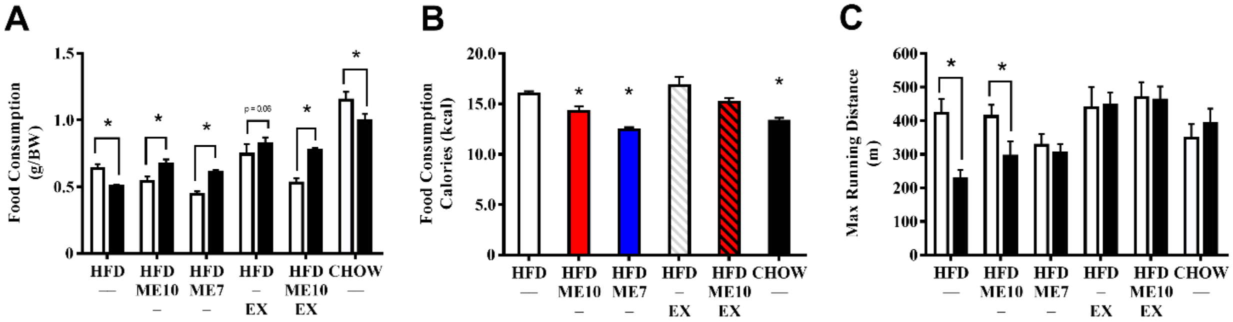

Figure S2. Experimental period food consumption and exercise performance. Food consumption per body weight (g/BW) was recorded (**A**) during the first week (open bars) and at the final week (filled bars) of experimental diet provision. Food consumption compared by two-way RM ANOVA, with comparisons only examined within group. *; $p<0.05$ compared to Pre (open bars) within group. (**B**) Mean daily food consumption (kcal) across the experimental diet period. One-way ANOVA with Fisher's LSD post-hoc test. *; $p<0.05$ compared to high-fat diet (HFD) group. (**C**) Aerobic capacity assessment via endurance stress test performed on animal treadmill, recorded prior to (Pre; open bars) and at the conclusion (Post, filled bars) of experimental periods. Running distance compared by two-way RM ANOVA, with comparisons only examined within group. *; $p<0.05$ compared to Pre (open bars) within group. Data are represented as mean ± SEM.

Supplementary Table S2. Antibody information

Antibody	Species	Source	Details	Primary	Secondary
Total OXPHOS	M	Abcam	ab110413	1:1000	goat anti-mouse IgG-HRP,1:10000
SOD1	R	Abcam	ab16831	1:1000	goat anti-rabbit IgG-HRP, 1:10000
SOD2	R	Abcam	ab13533	1:1000	goat anti-rabbit IgG-HRP, 1:10000
4-HNE	R	Abcam	ab46544	1:1000	goat anti-rabbit IgG-HRP, 1:10000
UCP1	R	Abcam	ab10983	1:1000	goat anti-rabbit IgG-HRP, 1:10000
AMPKα	R	Cell Signaling Technologies	2603S	1:1000	goat anti-rabbit IgG-HRP, 1:10000
Phospho-AMPKα (Thr172)	R	Cell Signaling Technologies	2531S	1:1000	goat anti-rabbit IgG-HRP, 1:10000

Table S2. Detailed information on primary and secondary antibodies and dilutions used for immunoblotting of muscle and white adipose tissue (WAT) homogenates. HRP; horseradish peroxidase, SOD1; Superoxide Dimutase 1, SOD2; Superoxide Dimutase 2, 4-HNE; 4-Hydroxynonenal, UCP1; uncoupling protein 1, AMPK; AMP-activated protein kinase.

Supplementary Table S3. Taqman Probes

Gene Symbol	Gene Name	Source	Details
**ADIPOQ**	Adiponectin, C1Q and collagen domain containing	Thermo Scientific	Mm00456425_m1
**CASP1**	Caspase 1	Thermo Scientific	Mm00438023_m1
**CIDEA**	Cell death inducing DNA fragmentation factor, alpha subunit-like effector A	Thermo Scientific	Mm00432554_m1
**COX2**	Cytochrome c oxidase subunit II	Thermo Scientific	Mm03294838_g1
**CPT1 ß**	Carnitine palmitoltransferase 1beta	Thermo Scientific	Mm00487191_g1
**CPT2**	Carnitine palmitoyltransferase 2	Thermo Scientific	Mm00487202_m1
**FATP1**	Solute carrier family 27 (fatty acid transporter), member 1	Thermo Scientific	Mm00449511_m1
**HSL**	Hormone sensitive lipase	Thermo Scientific	Mm00495359_m1
**IL-1ß**	Interleukin 1 beta	Thermo Scientific	Mm00434228_m1
**IL-6**	Interleukin 6	Thermo Scientific	Mm00446190_m1
**LCAD**	acyl-Coenzyme A dehydrogenase, long chain	Thermo Scientific	Mm00599660_m1
**MCAD**	acyl-Coenzyme A dehydrogenase, medium chain	Thermo Scientific	Mm01323360_g1
**PDK4**	Pyruvate dehydrogenase kinase	Thermo Scientific	Mm01166879_m1
**PGC1-α**	Peroxisome proliferative activated receptor, gamma, coactivator 1 alpha	Thermo Scientific	Mm01208835_m1
**PPAR-α**	Peroxisome proliferator activated receptor alpha	Thermo Scientific	Mm00440939_m1
**PRDM16**	PR domain containing 16	Thermo Scientific	Mm00712556_m1
**SCHAD (Hadh)**	Short-chain β-hydroxyacyl-Coenzyme A dehydrogenase	Thermo Scientific	Mm00492535_m1
**TNFA-α**	Tumor necrosis factor-alpha	Thermo Scientific	Mm00443258_m1
**UCP3**	Uncoupling protein 3 (mitochondrial, proton carrier)	Thermo Scientific	Mm01163394_m1

Table S3. Detailed Taqman Probes used in the examination of mRNA expression

Supplementary Experimental Procedures

Preparation of Permeabilized Muscle Fibers (PmFB) for mitochondrial respiration.

This technique is partially adapted from previous methods and has been described elsewhere [1–4]. In brief, small portions (~25 mg) of muscle were dissected from each excised muscle and placed in ice-cold BIOPS, containing (in mM): 50 MES, 7.23 K_2EGTA, 2.77 CaK_2EGTA, 20 imidazole, 0.5 dithiothreitol, 20 taurine, 5.77 ATP, 15 PCr, and 6.56 $MgCl_2·6 H_2O$ (pH 7.1). The muscle was trimmed of connective tissue and fat and divided into several small muscle 'bundles' (~2–7 mm, 2–5 mg wet weight). Each bundle was gently separated along the longitudinal axis with a pair of anti-magnetic needle-tipped forceps under magnification (Zeiss 2000, Germany). Bundles were then treated with 40 µg/mL saponin in BIOPS and incubated on a rotor for 30 min at 4°C. Saponin is a mild, cholesterol-specific detergent that selectively permeabilizes the sarcolemmal membranes while keeping mitochondrial membranes, which contain little cholesterol, intact [5,6]. Following permeabilization, the PmFB were washed in respiration medium (MiR05) containing (in mM): 0.5 EGTA, 10 KH_2PO_4, 3 $MgCl_2·6 H_2O$, 60 K-lactobionate, 20 Hepes, 20 Taurine, 110 sucrose and 1 mg/mL fatty acid free BSA (pH 7.1) until respiration experiments were initiated.

Mitochondrial Respiration in Permeabilized Muscle Fiber Bundles

High-resolution O_2 consumption measurements were conducted in 2 mL of MiR05 using the Oroboros Oxygraph-2k (Oroboros Instruments, Corp., Innsbruck, Austria). Experiments were conducted at an initial oxygen concentration of 350–375 µmol/L with constant stirring at 750 rev/min. Respiration medium contained 20 mM Cr to saturate mitochondrial creatine kinase [7] and 5 µM blebbistatin to prevent PmFB contraction [8,9]. For ADP-stimulated respiration, 5 mM pyruvate and 2 mM malate were added as complex I substrates (via generation of NADH to saturate electron entry into complex I), followed by a titration of submaximal ADP (25, 100 and 500 µM) and maximal ADP (5 mM). Succinate (20 mM) was then added under state III conditions to saturate electron entry into complex II. Complex II kinetics were determined through the stepwise addition of succinate in the presence of 5 mM ADP and 10 µM rotenone (to inhibit and prevent superoxide generation at Complex I). At the end of each experiment, cytochrome *c* was added to test for mitochondrial membrane integrity, with all experiments demonstrating < 10% increase in respiration [10]. Each protocol was completed before the oxygraph chamber [O_2] reached 150 µM. Polarographic oxygen measurements were acquired in 2 s intervals with the rate of respiration derived from 40 data points and expressed as pmol/s/mg dry weight.

Cytochrome *c* oxidase (COX), Citrate Synthase (CS) and short chain β-hydroxy-acyl-CoA-dehydrogenase (β-HAD) enzyme activity assays

Muscle was homogenized in Lysing Matrix D tubes (MP Biomedicals, Solon, OH, USA) using the FastPrep-24 Tissue and Cell Homogenizer (MP Biomedicals) for 5 x 5-second cycles at a speed of 4.0 m/s with samples placed on ice for 5 minutes between cycles. Samples were homogenized in 20 volumes of buffer containing 70 mM sucrose, 220 mM mannitol, 10mM HEPES, 1 mM EGTA, supplemented with protease inhibitors (Complete Mini®, Roche Applied Science, Laval, PQ, Canada). The maximal activities of citrate synthase (CS), cytochrome *c* oxidase (COX) and short chain β-hydroxy-acyl-CoA-dehydrogenase (β-HAD) were determined in homogenized SkM, as previously described [11,12]. For determination of CS maximal activity, 15 µL of muscle homogenate was added to cuvette containing: 825µL 0.1M Tris Buffer (pH 8.0), 100µl 5,5'-dithiobis (2-nitrobenzoic acid) (DTNB, 0.5mg/mL Tris Buffer) and 10 µl acetyl CoA (6mg/mL Tris Buffer). The cuvette was warmed to 37°C, and 50 µL of oxaloacetate (6.1mg/mL Tris buffer) was added to initiate the reaction. Absorbance was recorded at 412 nm for 120 seconds and the slope between 30 and 90 seconds was recorded. The maximal activities of cytochrome c oxidase (COX) was determined via methods previously described with small modifications [11].

Oxidized cytochrome *c* (Sigma C7752) was reduced by sodium dithionite in 0.05 M potassium phosphate buffer (KH_2PO_4, pH 7.4). Twenty microliters of muscle homogenate were added to 955 μL of 0.05 M potassium phosphate buffer and 30 μL of reduced cytochrome *c* in a cuvette that had been warmed to 37°C. The rate of oxidation of reduced cytochrome *c* was measured at 550 nm for 3 min at 37°C. For the determination of β-HAD, the following were added to a cuvette that had been warmed at 30°C: 800 μL of Tris buffer (Tris HCl 1 M, EDTA 200 mM; pH 7.0); 10 μL of Triton (10%) and 35 μL of homogenate was added and mixed. Following this, 10 μL of acetoacetyl CoA was added to initiate the reaction. The absorbance was recorded at 340 nm every 2 min for 4 min. Enzyme activity was expressed in nmol/min/mg protein. Assays have been previously established in the literature [11,12] were performed utilizing a spectrophotometer (Cary Bio-300, Varion, Inc., Palo Alto, CA, USA) that had been calibrated.

REFERENCES

1. Ydfors, M.; Hughes, M.C.; Laham, R.; Schlattner, U.; Norrbom, J.; Perry, C.G.R. Modelling in vivo creatine/phosphocreatine in vitro reveals divergent adaptations in human muscle mitochondrial respiratory control by ADP after acute and chronic exercise. *J. Physiol.* **2016**, *594*, 3127–3140, doi:10.1113/JP271259.
2. Tonkonogi, M.; Harris, B.; Sahlin, K. Mitochondrial oxidative function in human saponin-skinned muscle fibres: Effects of prolonged exercise. *J. Physiol.* **1998**, *510*, 279–286, doi:10.1111/j.1469-7793.1998.279bz.x.
3. Perry, C.G.R.; Kane, D.A.; Lanza, I.R.; Neufer, P.D. Methods for assessing mitochondrial function in diabetes. *Diabetes* 2013, *62*, 1041–1053.
4. Kuznetsov, A. V.; Thvel, T.; Sikk, P.; Kaambre, T.; Kay, L.; Daneshrad, Z.; Rossi, A.; Kadaja, L.; Peet, N.; Seppet, E.; et al. Striking differences between the kinetics of regulation of respiration by ADP in slow-twitch and fast-twitch muscles in vivo. *Eur. J. Biochem.* **1996**, *241*, 909–915, doi:10.1111/j.1432-1033.1996.00909.x.
5. Veksler, V.I.; Kuznetsov, A. V.; Sharov, V.G.; Kapelko, V.I.; Saks, V.A. Mitochondrial respiratory parameters in cardiac tissue: A novel method of assessment by using saponin-skinned fibers. *BBA - Bioenerg.* **1987**, *892*, 191–196, doi:10.1016/0005-2728(87)90174-5.
6. Kuznetsov, A. V.; Veksler, V.; Gellerich, F.N.; Saks, V.; Margreiter, R.; Kunz, W.S. Analysis of mitochondrial function in situ in permeabilized muscle fibers, tissues and cells. *Nat. Protoc.* **2008**, *3*, 965–976, doi:10.1038/nprot.2008.61.
7. Anmann, T.; Guzun, R.; Beraud, N.; Pelloux, S.; Kuznetsov, A. V.; Kogerman, L.; Kaambre, T.; Sikk, P.; Paju, K.; Peet, N.; et al. Different kinetics of the regulation of respiration in permeabilized cardiomyocytes and in HL-1 cardiac cells. Importance of cell structure/organization for respiration regulation. *Biochim. Biophys. Acta - Bioenerg.* **2006**, *1757*, 1597–1606, doi:10.1016/j.bbabio.2006.09.008.
8. Monaco, C.M.F.; Hughes, M.C.; Ramos, S. V.; Varah, N.E.; Lamberz, C.; Rahman, F.A.; McGlory, C.; Tarnopolsky, M.A.; Krause, M.P.; Laham, R.; et al. Altered mitochondrial bioenergetics and ultrastructure in the skeletal muscle of young adults with type 1 diabetes. *Diabetologia* **2018**, *61*, 1411–1423, doi:10.1007/s00125-018-4602-6.
9. Perry, C.G.R.; Kane, D.A.; Lin, C. Te; Kozy, R.; Cathey, B.L.; Lark, D.S.; Kane, C.L.; Brophy, P.M.; Gavin, T.P.; Anderson, E.J.; et al. Inhibiting myosin-ATPase reveals a dynamic range of mitochondrial respiratory control in skeletal muscle. *Biochem. J.* **2011**, *437*, 215–222, doi:10.1042/BJ20110366.
10. Kuznetsov, A. V.; Schneeberger, S.; Seiler, R.; Brandacher, G.; Mark, W.; Steurer, W.; Saks, V.; Usson, Y.; Margreiter, R.; Gnaiger, E. Mitochondrial defects and heterogeneous cytochrome c release after cardiac cold ischemia and reperfusion. *Am. J. Physiol. - Hear. Circ. Physiol.* **2004**, *286*,

doi:10.1152/ajpheart.00701.2003.
11. Carter, S.L.; Rennie, C.; Tarnopolsky, M.A. Substrate utilization during endurance exercise in men and women after endurance training. *Am. J. Physiol. - Endocrinol. Metab.* **2001**, *280*, doi:10.1152/ajpendo.2001.280.6.e898.
12. Nilsson, M.I.; Macneil, L.G.; Kitaoka, Y.; Alqarni, F.; Suri, R.; Akhtar, M.; Haikalis, M.E.; Dhaliwal, P.; Saeed, M.; Tarnopolsky, M.A. Redox state and mitochondrial respiratory chain function in skeletal muscle of LGMD2A patients. *PLoS One* **2014**, *9*, doi:10.1371/journal.pone.0102549.
